# Comparative analysis of femoral bone loss: uncemented vs. cemented aseptic stem loosening in first-time revision surgery—a retrospective evaluation of 215 patients

**DOI:** 10.1007/s00402-024-05506-z

**Published:** 2024-08-27

**Authors:** Nele Wagener, Sebastian Hardt, Matthias Pumberger, Friederike Schömig

**Affiliations:** Center for Musculoskeletal Surgery, Department of Orthopaedic Surgery, Charité Universitätsmedizin Berlin, Corporate member of Freie Universität Berlin and Humboldt-Universität Zu Berlin, Charitéplatz 1, 10117 Berlin, Germany

**Keywords:** Femoral bone defects, Paprosky classification, Aseptic loosening, Stem fixation (cemented/uncemented), First-time revision, Implant survival

## Abstract

**Introduction:**

The integrity of the femoral bone is crucial when considering reconstructive options for the first-time revision of a total hip arthroplasty (THA). Aseptic loosening of primary stems, whether cemented or uncemented, significantly affects the volume and quality of resultant femoral bone loss. This study evaluates the impact of the initial fixation method on femoral bone defect patterns by comparing the extent of bone loss.

**Materials and methods:**

A retrospective cohort of 215 patients with either cemented or uncemented stems, indicated for aseptic stem loosening, and undergoing first-time stem revision from 2010 to 2022 at our institution was analyzed. Femoral bone loss extent at first-time revision was preoperatively gauged using radiographs and categorized by the Paprosky classification. Survival probabilities pre-first-time revision for both stem types were calculated using Kaplan–Meier methods. Hazard ratios were applied to compare the risk of initial revision for uncemented versus cemented stems within the first and subsequent 2nd to 10th years post-primary implantation.

**Results:**

Cemented stems were associated with a higher occurrence of significant bone defects of type 3a (23.53% vs. 14.02%, p = .108), 3b (39.22% vs. 1.22%, p < .001), and 4 (3.92% vs. 0.00%) compared to uncemented stems. Conversely, smaller defects of type 1 and 2 were more prevalent in uncemented stem loosening (84.76% vs. 33.33%, p < .001). Notably, cemented stems exhibited a significantly prolonged revision-free period over the complete decade following primary insertion (p < .001). The unadjusted risk of first-time revision due to stem loosening showed a tendency to an increase in uncemented stems within the initial postoperative year (HR 5.55, 95% CI 0.74; 41.67, p = .096), and an adjusted risk of 2.1 (95% CI 0.26; 16.53, p = .488). However, these differences did not reach statistical significance. In the subsequent 2nd-10th years, the risk was lower compared to cemented stems (HR 2.35, 95% CI 1.39; 3.99, p = .002).

**Conclusions:**

Uncemented primary stems necessitating first-time revision due to aseptic loosening demonstrated notably smaller femoral bone defects in comparison to primary cemented stems.

## Introduction

As advancements in total hip arthroplasty (THA) continue to enhance surgical outcomes, the demand for these procedures is expected to rise significantly. This trend correlates with an aging demographic and a corresponding increase in osteoarthritic conditions that require orthopedic intervention. Consequently, the surgical landscape is evolving to accommodate a higher volume of THA implantations, driven by clinical necessity and the procedural success rates [1]. Notably, a common sequela following THA implantation is periprosthetic bone loss, which can complicate subsequent interventions [2, 3]. In the foreseeable future, orthopedic surgeons will increasingly encounter intricate scenarios of bone loss as the frequency of revision and post-revision procedures escalates. Although THA is deemed a safe intervention, a non-negligible segment of patients necessitates revision surgeries. Predominant reasons for femoral revision post-THA include aseptic loosening [4–6] periprosthetic infection [4, 7] fracture [4], dislocation [4], malpositioning of the implant, metallosis [8] and implant breakage [4, 5]. The complexity of femoral revisions after primary THA is compounded by the quantity and quality of bone loss, which is influenced by a myriad of factors: the mode of stem fixation, stress shielding, surgical technique, surgeon's experience and preference, patient-specific factors, extensive osteolysis, and pronounced loosening [9].

The debate around the preferred type of stem fixation for primary THA persists, with the trend leaning towards uncemented stem implantation, particularly in younger patients [10]. Despite the notable success rates of both uncemented and cemented THA systems, implant failure remains a critical complication, prompting revision surgery. These failures are often coupled with significant bone loss, which poses challenges for further revision and fixation procedures [11]. A study indicated that the revision rate for uncemented THAs is twice that of the cemented THAs at five years [12]. In another study, the uncemented group showed significantly higher complication rates, including radiographic loosening, periprosthetic fractures, dislocation, and heterotrophic ossification. The revision rates were significantly higher in the uncemented group (66/444) compared to the cemented group (27/441) [13]. Babazadeh et al. stated that the cumulative revision rate (CPR) at 17 years was higher for cementless femoral prostheses (10.5%, 95% CI 8.4–13.1) compared to cemented polished tapered femoral prostheses (6.4%, 95% CI 6.0–6.8) [14].

The revision rate for fully cemented THAs was found to be lower than that for uncemented THAs, especially in patients over 65 years of age [15]. Another study highlighted that the prosthetic loosening rates for cemented (CE) and uncemented (UN) groups were 16.8% and 26.4%, respectively, at a minimum of 5 years. The early revision rate was significantly lower for the cemented group (7.6% CE) compared to the uncemented group (14.8% UN) [16].

In contrast, Hooper et al. have demonstrated a lower rate of aseptic loosening with uncemented femoral stems in primary THA, particularly among younger patients [17]. These findings suggest that while cemented THAs typically have lower revision rates for all causes in the short term (90 days), uncemented THAs exhibit a significantly lower rate of aseptic loosening in individuals under 65 years of age. Furthermore, Wechter et al. support this notion, indicating that uncemented stem components are associated with a reduced risk of stem revision due to aseptic loosening compared to their cemented counterparts [18]. This evidence suggests a potential preference for uncemented stems in younger demographics and highlights the complexity in choosing the optimal stem fixation method for THA.

As advancements in THA continue to enhance surgical outcomes, increasing attention is being paid to the underlying factors contributing to post-surgical complications such as aseptic loosening, a leading cause of THA failure and revision surgery.

However, registry studies often do not take into account the extent of femoral bone defects, which may affect both the choice of fixation method and revision rates. In contrast, observational studies have shown that for comparable femoral bone defects, the fixation method has no obvious influence on implant survival [19]. The decision-making process for fixation methods in primary THA is complex and includes scientific evidence, individual surgeon preferences and patient-specific factors [20]. For accurate assessment of bone loss and to delineate the morphology of the residual proximal femoral bone, the Paprosky classification system was introduced in 1997 (Table 1). The objective of this study is to stratify the risk relative to the size of femoral bone defects during first-time revision by analyzing the influence of cemented versus uncemented fixation, indications for primary and revision THA, pre-existing medical conditions, and demographic data in cases of aseptic stem loosening.

## Materials and methods

This study received approval from the institutional ethics committee (EA4/129/23). We conducted a retrospective review, encompassing all patients who underwent primary total hip arthroplasty (THA) and subsequent first-time revision due to aseptic stem loosening at our institution from January 2010 to December 2022.

Using structured inclusion and exclusion criteria, out of 1,365 initial revisions, 215 cases were selected that involved aseptic loosening of both cemented and uncemented stems. Exclusion criteria for the study ruled out cases involving first-time revisions of THA due to: (1) acetabular loosening (n = 313), (2) incomplete patient data (n = 265), (3) periprosthethic fractures (n = 182), (4) replacement of head or inlay, metallosis (n = 175), (5) hip dislocation (n = 55), (6) painful THA (n = 53), (7) pelvitrochanteric insufficiency, psoas impingement, prosthesis impingement (n = 37), (8) leg length discrepancy (n = 11), (9) poor X-ray quality (n = 8), (10) THA dislocation (n = 5), (11) implant fracture/failure (n = 4), (12) instability (n = 2), and (13) septic stem-loosening (n = 40). Data collection leveraged electronic health records, focusing on (1) the duration of implant survival, (2) indications for both primary and revision THA, (3) the extent of Paprosky femoral bone defects, and (4) patient comorbidities.

### Bone defect size

**Fig. 1:**
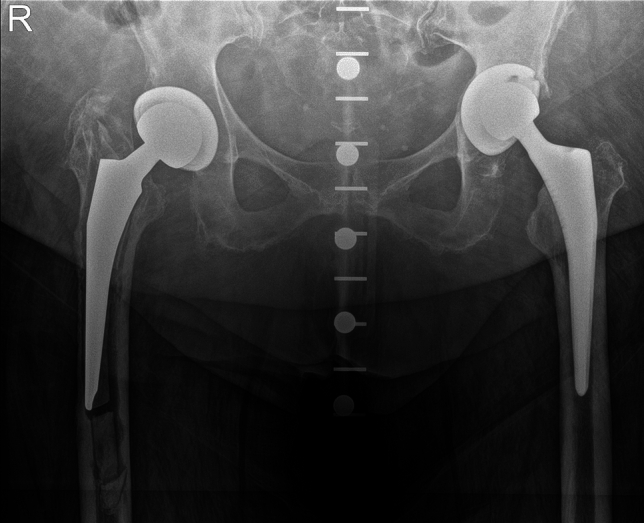
shows a patient with aseptic loosening of the right cemented primary stem and a significant femoral defect situation classified as Paprosky 3a, who underwent a stem revision

Preoperative radiographic assessments conducted at the time of the first revision, including pelvic overviews and axial hip images, were reviewed. Two independent investigators, NW and SH, classified the femoral bone defect dimensions based on the Paprosky criteria (Table 1). In cases where consensus between NW and SH was not reached, a third independent chief senior surgeon was consulted. For each patient, the pre-revision femoral bone loss was categorized in accordance with the system established by Paprosky et al. utilizing operative reports and pre-revision pelvic radiographs.The Paprosky classification was the metric of choice due to its widespread application in evaluating femoral bone defects in preceding research. Additionally, osteoporosis was considered and evaluated through patient medical histories, and radiographic evidence indicative of osteoporosis. In the context of our study, the evaluation of osteoporosis prevalence at the time of primary implantation revealed no significant differences between the primary cemented and uncemented stem fixation groups within our cohort (Fig. 1).

**Table 1:** femoral bone defect size according to Paprosky et al

I	Minimal metaphyseal bone loss and preserved diaphyseal structure
II	Balloted or funnel-shaped bony changes metaphyseal with intact diaphyseal structure
III	Extensive bone loss metaphyseally extending to the diaphysis
IIIa	> 4 cm intact bony structure at the isthmus femoris
IIIb	< 4 cm intact bony structure at the level and distal to the isthmus femoris
Iv	Extensive bone loss metaphyseal and diaphyseal with wide medullary canal; No remaining isthmus femoris

### Statistics

Categorical data are reported as frequencies and percentages, while continuous data are presented as means with standard deviations. The chi-square test or Fisher’s test was used to validate the association (p < 0.05) in the bivariate analysis. For continuous variables test of the normality (Kolmogorov–Smirnov) is performed for deciding the measures of central tendency and statistical methods for data analysis. Data were called as normally distributed if p > 0.05. When data follow normal distribution, mean and standard deviation are reported and parametric tests (t-Test or ANOVA) are used otherwise median and quartiles are reported and nonparametric methods (Mann–Whitney-U test, Kruskall-Wallis test) are applied. Survival analysis was conducted using the Kaplan–Meier method, and a Cox proportional hazards model was employed to adjust for potential confounding variables. All statistical tests were two-tailed and maintained a significance threshold of 5%. Statistical analyses were performed using SPSS version 29, IBM Inc. and R (survival analysis).

## Results

**Fig. 2:**
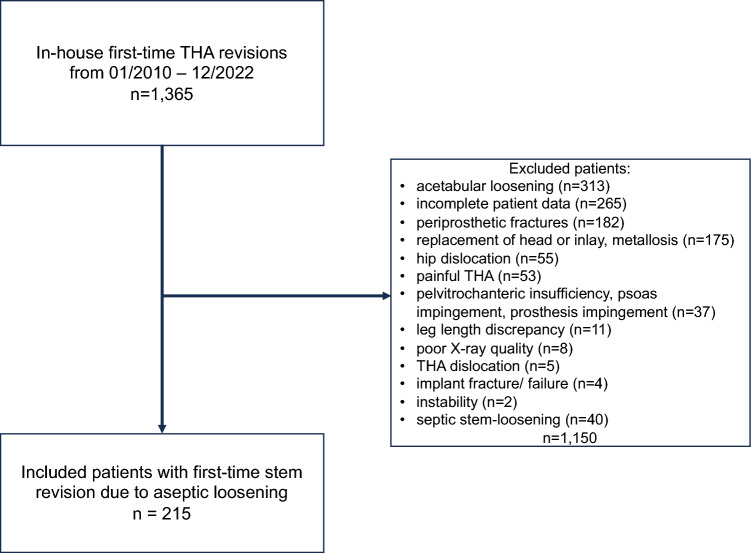
Flow-chart of patient selection

**Fig. 3:**
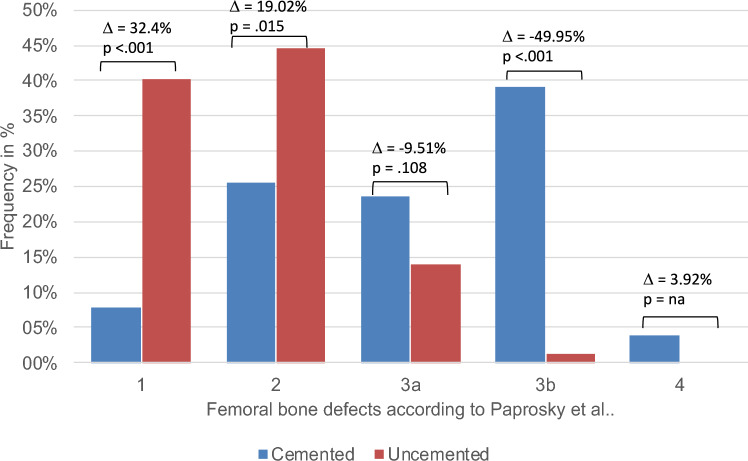
Bone defect size between cemented and uncemented stems

The patient selection process is illustrated in Fig. 2. The study encompassed 215 patients (122 females, 93 males) with an average age of 74 years at the time of their first revision surgery Fig. 3. The cohort was divided into two groups based on the type of stem fixation: 51 patients (23.72%) with aseptic loosening of a cemented stem and 164 patients (76.28%) with stem loosening of an uncemented stem. Demographic details and clinical characteristics of the patients are presented in Tables 2 and 3.

**Table 2:** Study population characteristics

		Cemented	Uncemented	*p* value
		*N* = 51	*N* = 164	
Age		58.10 ± 16.83	57.96 ± 12.38	0.484
Sex	Female/Male	39/12 (76.47/23.53%)	83/81 (50.61/49.39%)	0.001
BMI	> 30	10 (19.6%)	55 (33.54%)	0.080
ASA	1	3 (5.88%)	24 (14.63%)	0.055
	2 + 3	47 (92.16%)	140 (85.37%)	
	4	1 (1.96%)	0	
CHD	Yes/No	31/20 (60.78%)	78/86 (47.65%)	0.099
COPD	Yes/No	4/47 (7.84%)	10/154 (6.10%)	0.659
Gastric ulcera	Yes/No	2/49 (3.92%)	1/163 (0.61%)	0.078
Liver disease	Yes/No	3/48 (5.88%)	11/153 (6.71%)	0.835
Apoplexy	Yes/No	2/49 (3.92%)	5/159 (3.05%)	0.759
Dementia	Yes/No	1/50 (1.96%)	1/163 (0.61%)	0.380
PAD	Yes/No	1/50 (1.96%)	3/161 (1.83%)	0.952
Diabetes mellitus	Yes/No	4/47 (7.84%)	18/146 (10.98%)	0.519
Oncological disease	Yes/No	6/45 (11.76%)	23/141 (14.02%)	0.680
Renal failure	Yes/No	4/47 (7.84%)	18/146 (10.98%)	0.519
Rheumatoid arthritis	Yes/No	2/49 (3.92%)	7/157 (4.27%)	0.914
Hypothyroidism	Yes/No	6/45 (11.76%)	22/142 (13.41%)	0.760
AIDS	Yes/No	0/51 (0.00%)	1/163 (0.61%)	NA
Smoking	Yes/No	9/42 (17.65%)	21/143 (12.80%)	0.383
Alcohol	Yes/No	6/45 (11.76%)	18/146 (10.98%)	0.876
Osteoporosis	Yes/No	10/41	16/148	0.083
Diagnosis at primary THA	Primary osteoarthritis	43 (84.31%)	137 (83.54%)	0.896
	Secondary osteoarthritis	8 (15.69%)	27 (16.46%)	0.896

Demographics for metric variable age and BMI are presented as mean ± standard deviation, for categorical variables as total numbers and frequencies

*PAD* Peripheral arterial disease, *CHD* coronary heart disease, *COPD* chronic obstructive pulmonary disease, *ASA* American Society of Anesthesiologists, *THA* Total hip arthroplasty

*p* values resulting from Chi-Square test or Fisher exact test for categorical variables and Mann–Whitney test for age. *NA* not applicable due to zero cell frequencies

**Table 3:** Characteristics of first-time revision

		Male			Female			Total		
		Cemented *n* = 12	Uncemented *n* = 81	*p* value	Cemented *n* = 39	Uncemented *n* = 83	*p* value	Cemented	Uncemented	*p* value
Age at 1st time revision		80 (70; 84)	70 (63; 75)	0.009	77 (71; 83)	70 (61; 77)	< 0.001	78 (71; 83)	70 (63; 76)	< 0.001
BMI		27.9 (24.6; 31.1)	29.1 (26.2; 31.4)	0.399	25.6 (23.3; 28.0)	26.8 (24.1; 30.1)	0.017	25.7 (23.4; 29.3)	28.0 (24.9; 30.9)	0.011
Implant survival (mo.)		195 (138; 288)	85 (36; 146)	0.001	180 (62; 336)	104 (48; 154)	0.007	180 (63; 324)	93 (43; 150)	< 0.001
Stem loosening		7 (58.3%)	59 (72.8%)	0.302	16 (41.0%)	52 (62.7%)	0.025	23 (45.1%)	111 (67.7%)	0.004
Stem and cup loosening		5 (41.7%)	22 (27.2%)	0.302	23 (59.0%)	31 (37.3%)	0.025	28 (54.9%)	53 (32.3%)	< 0.001
Type of revision	Stem replacement	7 (58.3%)	47 (58.0%)	0.984	10 (25.6%)	44 (53.0%)	0.005	17 (33.3%)	91 (55.5%)	0.006
	THA replacement	5 (41.7%)	34 (42.0%)	0.984	29 (74.4%)	39 (74.0%)	0.005	34 (66.7%)	73 (44.5%)	0.006
Surgery duration (min.)		166 (148; 191)	123 (104; 167)	0.005	163 (125; 242)	119 (92; 159)	< 0.001	165 (132; 235)	122 (98; 167)	< 0.001

Comparison of parameters of first-time revision between cemented and uncemented stems. Demographics for metric variables (normally distribution cannot be assumed for any variable) are presented as median (lower; upper quartile), for categorical variables as total numbers and frequencies

*BMI* body-mass-index (kg/m^2^), *mo.* Months, *THA* Total hip arthroplasty

*p* values resulting from Chi-Square test or Fisher exact test for categorical variables and Mann–Whitney test for age. *NA* not applicable due to zero cell frequencies

The mean age at the time of primary surgery for both the cemented and uncemented stem loosening groups did not differ significantly (58.10 vs. 57.96 years, p = 0.484). However, the proportion of females is higher in the cemented group than in the uncemented group (76.47% vs. 50.61%, p = 0.001). To account for this imbalance gender is used as an adjusting factor in the subsequent analyses. Notably, within the cemented stem group, a slightly higher proportion of patients was classified with an ASA score of 2–3, in contrast to patients in the uncemented stem group with the same ASA classification at the time of first revision (92.16% vs. 85.37%, p = 0.055). The incidence of coronary heart disease (CHD) was observed to be more frequent in patients with cemented stem loosening compared to those with uncemented stem loosening, but this difference was not statistically significant (60.78% vs. 47.65%, p = 0.099). The initial diagnosis of primary osteoarthritis (84.31% vs. 83.54%, p = 0.896), followed by secondary osteoarthritis (15.69% vs. 16.46%, p = 0.896), was similarly distributed between cemented and uncemented stems at the time of primary surgery.

### First-time revision due to aseptic stem loosening

During the initial revision period, the incidence of aseptic stem loosening was found to be more frequent in uncemented stems compared to cemented stems (67.68% vs. 45.10%, p = 0.004). In contrast, patients with cemented stems showed concurrent cup loosening more frequently than patients with uncemented stems (54.90% vs. 32.32%, p < 0.001) (Table 3). On average, the need for revision of cemented stems arose significantly later in comparison to uncemented stems (180 vs. 93 months, p < 0.001). Furthermore, the average duration of surgical intervention for the first-time revision of cemented stems exceeded that of uncemented stems (165 vs. 122, p < 0.001). Gender specific differences could be observed for BMI, where female showed a significant BMI difference between the cemented and uncemented group (p = 0.017), stem loosening (p = 0.025, only for female), stem and cup loosening (p = 0.025, only for female), type of revision (p = 0.005, only for female).

### Comparative analysis of revision stems for cemented and cementless fixation

The principles for revision choice include considerations of bone quality, patient-specific factors, defect classification, and detailed preoperative planning. Bone quality influences the choice between cementless and cemented stems, especially in cases of extensive bone loss or osteoporosis. Patient-specific factors, such as overall health, comorbidities, previous surgeries, activity level, and life expectancy, are crucial. Defect classification according to systems like Della Valle and Paprosky guides implant choice, with more severe defects requiring complex solutions. Detailed preoperative planning and the preparation of special instruments are essential to anticipate surgical challenges. The choice of surgical approach and techniques is based on individual case scenarios and intraoperative findings [21, 22]. Specifically, when comparing initially cemented versus uncemented stems, there was a preference for different revision implants: 33.33% of the primarily cemented stems were revised with a cementless Revitan revision stem, as opposed to 14.02% of the initially uncemented stems (p = 0.002). In contrast, 23.53% of the primarily cemented stems received a cemented VerSys stem, compared to 0.61% of the uncemented group (p < 0.001). Additionally, 17.65% of the initially cemented stems were revised using a cementless SLR stem, against 31.10% in the uncemented cohort, but this difference was not statistically significant (p = 0.061) (Table 4). Furthermore, within the group of primarily uncemented stems, 38% received an uncemented SL-Plus-MIA stem during the first-time revision procedure (38.41% vs. 3.92%, p < 0.001).

**Table 4:** Revision arthroplasty characteristics

		Cemented stem in primary surgery	Uncemented stem in primary surgery		
		*N* (%)	*N* (%)	*p* value	*p* value (overall)
Revision stem brand	Revitan (uncemeted)	17 (33.33%)	23 (14.02%)	0.002	< 0.001
	VerSys (cemented)	12 (23.53%)	1 (0.61%)	< 0.001	
	SLR (uncemeted)	9 (17.65%)	51 (31.10%)	0.061	
	SPII Lubinus (cemented)	8 (15.69%)	3 (1.83%)	< 0.001	
	SL-Plus-MIA (uncemeted)	2 (3.92%)	63 (38.41%)	< 0.001	
	Alloclassic Zweymüller (uncemeted)	2 (3.92%)	17 (10.37%)	0.157	
	Megasystem-C (uncemented)	1 (1.96%)	0%	NA	
	Wagner SL (uncemented)	0	4 (2.44%)	NA	
	Bicontact (cemented/uncemented)	0	1 (0.61%)	NA	
	TRJ (uncemented)	0	1 (0.61%)	NA	

*p* values resulting from Chi-Square test. *NA* not applicable due to zero cell frequencies

### Increased femoral bone loss associated with primary stem cementation

According to the Paprosky classification, cemented stems were associated with substantial femoral bone defects—type 4 and 3b—in 43.14% of cases, in contrast to 1.22% observed in uncemented stems (p < 0.001) (Table 5). Furthermore, type 3a femoral bone defects was found significantly more frequently in patients with cemented stems compared to patients with uncemented stems (23.53% vs. 14.02%, p = 0.108). On the other hand, uncemented stems were significantly more often associated with the smaller type 1 and 2 defects than cemented stems were (84.76% vs. 33.33%, p < 0.001). Differences can be seen both in female and male.

**Table 5:** Size of femoral bone defects at the time of first-time revision

		Male			Female			Total			
		Cemented *N* (%)	Uncemented *N* (%)	*p* value	Cemented *N* (%)	Uncemented *N* (%)	*p* value	Cemented *N* (%)	Uncemented *N* (%)	*p* value	*p* value (overall)
Femoral bone defects according to Paprosky et al	1 and 2	3 (25.0%)	70 (86.4%)	0.001	14 (35.9%)	69 (83.1%)	< 0.001	17 (33.3%)	139 (84.78%)	< 0.001	< 0.001
	3a	4 (33.3%)	10 (12.3%)		8 (20.5%)	13 (15.7%)		12 (23.5%)	23 (14.0%)	0.108	
	3b and 4	5 (41.7%)	11(1.2%)		17 (43.6%)	1 (1.2%)		22 (43.1%)	2 (1.2%)	< 0.001	
	1	0 (0%)	35 (43.2%)	< 0.001	4 (10.3%)	31 (37.3%)	< 0.001	4 (7.8%)	66 (40.2%)	< 0.001	< 0.001
	2	3 (25.0%)	35 (43.2%)		10 (25.6%)	38 (45.8%)		13 (25.5%)	73 (44.5%)	0.015	
	3a	4 (33.3%)	10 (43.2%)		8 (20.5%)	13 (15.7%)		12 (23.5%)	23 (14.0%)	0.108	
	3b	4 (33.3%)	1 (1.2%)		16 (41.0%)	1 (1.2%)		20 (39.2%)	2 (1.2%)	< 0.001	
	4	1 (8.3%)	0 (0%)		1 (2.6%)	0 (0%)		2 (3.9%)	0 (0%)	NA	

*p* values resulting from Chi-Square test. *NA* not applicable due to zero cell frequencies

### Cemented stems exhibit superior longevity free from revision surgery

**Fig. 4:**
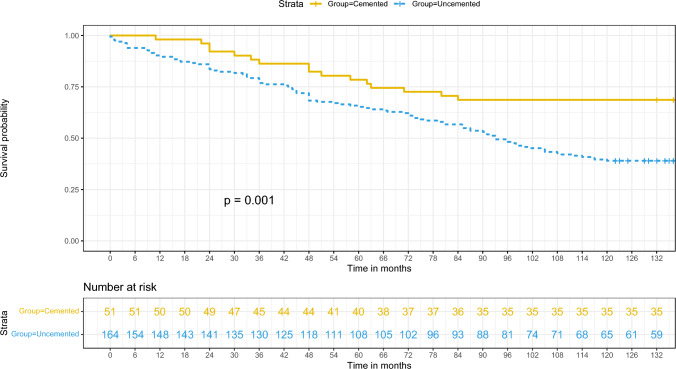
Survival rate of the stem depending on the type of fixation until first-time revision with the endpoint aseptic stem loosening

The ten-year survival rate of implants, unadjusted for confounding variables and with the endpoint being first-time revision for aseptic stem loosening, was significantly superior with cemented primary stems compared to uncemented ones (0.69; 95% Confidence Interval [CI] 0.56–0.81 vs. 0.39; 95% CI 0.32–0.46; see Fig. 4) (p < 0.001). In the initial two years following primary implantation, the survival probability, when adjusted for covariates, was greater in patients with cemented stems than those with uncemented stems, who demonstrated an elevated risk of aseptic stem loosening necessitating first-time revision.

During the interim period of two to eight years post-implantation, the adjusted risk for requiring a first-time revision due to aseptic loosening was increased in the uncemented stem cohort; however, the confidence intervals were overlapping, indicating statistical uncertainty. Commencing at the eighth year subsequent to the primary implantation, the adjusted survival probability for cemented stems markedly surpassed that of uncemented stems.

Kaplan Meier (KM) curves (shaded area indicate the 95% CI) and Log-rank test for survival rate of the stem over grouped factors. The KM survival curves for each grouped factor were identified by color and pattern differences.

### Risk of aseptic stem revision

The risk of first-time aseptic stem revision for uncemented stems was notably higher, presenting a 5.5-fold increase within the initial year following primary implantation, compared to cemented stems (Hazard Ratio [HR] 5.5; 95% Confidence Interval [CI] 0.7–41.7, p = 0.096) as detailed in Table 6. Adjusting for sex the adjusted one year HR for uncemented vs cemented is 4.78 (95% Confidence Interval [CI] 0.6–36.5, p = 0.132) and with respect to other covariates 2.08 (95% Confidence Interval [CI] 0.3–16.5, p = 0.488). However, this elevated risk diminishes over time, with the hazard of first-time revision from the second to the tenth year post-implantation being 2.4 times higher for uncemented stems in contrast to cemented stems (HR 2.4; CI 1.39–3.99, p = 0.002) unadjusted, 2.67 (1.3–3.9, p = 0.003) adjusted for sex and HR 2.1 (0.26–16.53, p = 0.488 adjusted for sex, age and Paprosky classes).

**Table 6:** Hazard ratio (HR) for first-time revision due to aseptic stem loosening of cemented and uncemented primary stems

	Model 1 HR (95% CI) *p* value		Model 2 HR (95% CI) *p* value		Model 3 HR (95% CI) *p* value	
2–10 year revision						
Uncemented vs. Cemented	2.35 (1.39; 3.99)	0.002	2.67 (1.33; 3.87)	0.003	1.69 (0.89; 3.21)	0.106
Female vs. Male	__		0.84 (0.58; 1.22)	0.359	0.84 (0.58; 1.22)	0.363
Age	__					0.046
Paprosky classes					0.45 (0.24; 0.85)	0.014
IIIA vs. I and II					0.73 (0.31; 1.73)	0.473
IIIB and IV vs. I and II					0.98 (0.97; 1.00)	0.056
1 year revision						
Uncemented vs. Cemented	5.55 (0.74; 41.67)	0.096	4.78 (0.63; 36.54)	0.132	2.08 (0.26; 16.53)	0.488
Female vs. Male			0.58 (0.22; 1.50)	0.258	0.58 (0.22; 1.52)	0.271
Age						0.500
Paprosky classes					0.29 (0.04; 2.26)	0.239
IIIA vs. I and II					0 (na)	0.980
IIIB and IV vs. I and II					1.00 (0.95; 1.04)	0.815

Regression was performed unadjusted (model 1), adjusted for sex (model 2) and adjusted for age, sex and femoral bone defect size (aggregating defects into 3 main groups along Paprosky classes 1 + 2, 3a, 3b + 4, model 3). Cox-regression hazards ratio (HR) in multivariate analysis for predicting time to first-time revision due to aseptic stem loosening

## Discussion

Our investigation into initial revisions after primary THA has demonstrated that cemented stems with aseptic loosening present with substantially larger femoral bone defects, classified as type 3a, 3b and 4 according to Paprosky et al., compared to uncemented stems. We observed that at primary implantation, 76.28% of the stems used were uncemented. It's worth mentioning that the choice between cemented and uncemented femoral stems varies around the world [23, 24]. This variation usually mirrors patient-specific requirements [24] local clinical practices and the preferences for certain procedures [23, 24].

Direct comparative analyses focusing on the survival of uncemented and cemented femoral fixation, particularly considering femoral bone defects, are scant. The choice of fixation remains a contentious topic within the orthopedic community [25]. Tyson and colleagues analyzed bone defects in the femur after re-revision and reported that the cementless fixation had a bone defect size of 3a, while the cemented group had more severe bone defects [19]. Our findings are in concurrence with Tyson et al., indicating that cemented fixations are prone to more severe bone defects of types 3b-4 (43.14% vs. 1.22%, p < 0.001). The cemented hip stems rely on bone cement (methyl methacrylate) to secure the stem to the bone, which, over time, may loosen, leading to bone damage and larger defects [26].

It is hypothesized that the patient's biological response to the presence of bone cement might lead to attempts to circumvent or degrade the cement, resulting in amplified defects [27, 28]. Material abrasion from peri-implant osteolysis in cemented hip THA can release particulate matter, eliciting an immune response and prompting osteoclastogenesis, leading to aseptic inflammation and peri-implant bone loss [29]. Furthermore, the application of bone cement can exert undue pressure on the bone, potentially leading to erosion or weakening. However, bone defect development is also contingent upon other factors, including patient's bone quality, comorbidities, and surgical technique [30].

The prevalence of severe bone defects of types 3a, 3b, and 4 post-primary stem cementation is corroborated in our study cohort by the selection of revision implants at the time of first revision. Primarily cemented stems necessitated modular, distal-fixating Revitan stems in 33.33%, cemented VerSys-long revision stems in 23.53%, and cemented SPII Lubinus long revision stems in 15.69% to manage severe femoral bone defects in the revision surgery. This underscores the correlation between the incidence of larger bone defects at initial revision and the employment of cement. Conversely, smaller type 1 and 2 femoral bone defects in our cohort with primarily uncemented stems were managed using SL-Plus MIA stems with press-fit fixation in 38.41% and SLR stems with press-fit fixation in 31.10%, following minimally invasive implantation techniques. In our cohort, the choice of the revision stem was primarily determined by patient-specific factors such as bone quality, the size of the bone defect, and the patient's health condition; however the influence of surgeon preference cannot be excluded.

Our analyses indicated that implant longevity until first-time revision was on average 7.25 years greater for cemented stems than for uncemented ones, with uncemented stems displaying a higher revision rate within the first two years post-primary implantation. In a 2021 study, Tyson et al. found that during the first three years after hip revision surgery, uncemented stems had a higher risk of re-revision than cemented stems, however, this difference diminished after four years [19]. Notably, our data also revealed a non-significant trend towards an increased risk of first-time revision during the first postoperative year with uncemented stems. Given the more extensive bone damage in cemented stems and their longer revision-free interval, it becomes crucial to consider the patient's age at primary implantation when selecting the fixation method.

Our research recorded that patients, whether with cemented or uncemented primary stems, had an almost equal age of 58 years at the time of primary implantation. At the time of first-time revision, the average age was 78 years for patients with cemented stems and 70 for those with uncemented fixation. In older patients, this may tip the balance in favor of a cemented stem, offering reduced revision risk and increased implant longevity. Cemented hip stems are favored for patients with lower bone quality, often in older individuals or those with osteoporosis, providing safer and more stable options, especially in revision surgeries and for those with complex hip anatomy (Dorr B, C). Our findings demonstrate that although cemented stems are associated with more severe bone defects at the time of revision compared to uncemented stems, they also show a prolonged revision-free period. This observation may suggest that the increased survival of cemented implants allows for larger bone defects to develop before revision surgery becomes necessary. One possible interpretation of this pattern could be that the durable bond provided by cemented fixation might tolerate greater bone loss, delaying the clinical need for revision. This durability potentially allows for a longer period during which bone defects can accumulate without impacting the stability of the implant significantly enough to require immediate revision. However, while this interpretation highlights a potential advantage of cemented fixation in terms of longevity, it also underscores the importance of careful monitoring and management of bone health in patients with cemented stems to mitigate the risk of severe bone loss over time. In younger patients, the emphasis should be on bone-sparing fixation and the preservation of an adequate bone reserve for future revisions.The choice between cemented and uncemented stems also depends on the surgeon's experience and preference, emphasizing a personalized approach based on each patient's specific health needs.

Previous studies have shown that several comorbidities such as diabetes mellitus, hypoalbuminemia, obesity, deficiency anemia, liver disease, peripheral vascular diseases, cancer, and psychotic disorders influence the revision risk after total hip and knee arthroplasty [31–33]. We observed that patients with cemented stems had increased coronary heart disease before first-time revision. It is hypothesized that the observed association between the higher prevalence of coronary heart disease at the time of the first revision and the longer implant survival of cemented stems may be related to the advanced age of the patients.

Our study has some limitations, including the lack of complete comparability of baseline data between groups, such as a higher proportion of females in the cemented group. To address these differences and consider the gender data gap, we stratified the results by gender. There were no significant differences in the prevalence of comorbid conditions between the groups, which strengthens the comparability of the results. However, to our knowledge, this is the first study to directly compare the effect of fixation on femoral bone defect size at the time of first-time revision after primary THA, revision risk, and implant longevity in aseptically loosened stems.

In summary, in our cohort, uncemented primary stems show significantly less severe femoral bone defects at first-time revision. Preservation of the bone stock is essential because future revision surgeries are expected given demographic trends especially in young patient age. This approach allows more healthy bone to be available for stem anchorage in subsequent revisions.

Our analyses showed that cemented primary stems revised for aseptic stem loosening had significantly greater femoral bone defects at the time of first-time revision, but with longer revision-free implant survival. Therefore, primary stem cementation may be considered in elderly patients with short life expectancy after weighing patient-specific risk factors.

## Conclusions

Uncemented primary stems revised for the first time due to aseptic stem loosening show significantly less severe femoral bone defects than primary cemented stems.

## Data Availability

The data underlying this study are available from the corresponding author upon reasonable request.

## Abbreviations

**THA**: Total hip arthroplasty
**CE**: Cemented
**UN**: Uncemented
**CI**: Confidence interval
**CHD**: Coronary heart disease
**BMI**: Body mass index
**ASA**: American society of anesthesiologists
**PAD**: Peripheral arterial disease
**COPD**: Chronic obstructive pulmonary disease
**NA**: Not applicable
**HR**: Hazard ratio
